# Bronchoscopy in COVID-19 inpatients: experience of a university hospital in the first outbreak of the disease in Brazil

**DOI:** 10.31744/einstein_journal/2022AO6858

**Published:** 2022-05-20

**Authors:** Sergio Eduardo Demarzo, Júlia Bamberg Cunha Melo, Mariasol Ximena Martínez Carranza, Felipe Nominando Diniz Oliveira, Anarégia de Pontes Ferreira, Addy Lidvina Mejia Palomino, Viviane Rossi Figueiredo, Marcia Jacomelli

**Affiliations:** 1 Universidade de São Paulo Faculdade de Medicina Hospital das Clínicas São Paulo SP Brazil Instituto do Coração (Incor), Hospital das Clínicas, Faculdade de Medicina, Universidade de São Paulo, São Paulo, SP, Brazil.

**Keywords:** Bronchoscopy, Bronchoalveolar lavage, COVID-19, Coronavirus infections, SARS-CoV-2, Betacoronavirus, Intubation, intratracheal, Respiration, artificial

## Abstract

**Objective:**

To describe the indications and endoscopic findings of bronchoscopy performed at a reference university hospital for inpatients diagnosed with COVID-19 during the first outbreak of the disease in Brazil.

**Methods:**

A retrospective analysis of medical records of adult patients diagnosed with COVID-19 who underwent bronchoscopy at the intensive care units of *Instituto do Coração* and *Hospital das Clínicas da Faculdade de Medicina da Universidade de São Paulo*, from March to August 2020.

**Results:**

A total of 132 bronchoscopies were performed in 103 patients diagnosed with COVID-19. Mean age was 56.1±14.5 years, and distribution was similar in both sexes. More than one test was performed in 16 patients. The most frequent indications were diagnostic endoscopic evaluation and therapeutic procedures in 78.6% of cases (n=81) and material collection in 21.4% of cases (n=22). The most frequent endoscopic findings were presence of secretion or clots in 34% of cases, the presence of acute inflammatory changes in 22.3%, and tracheal wall laceration in 20.4%. In 27.2% of patients, no relevant bronchoscopic findings were observed. In three patients, bronchoscopy was indicated to assess hemoptysis, but there was only one case of active bleeding. Procedure-related complications were not observed in this group of patients.

**Conclusion:**

Bronchoscopy proved to be a safe and effective procedure to assist in treatment of COVID-19 patients, and the most frequent indications were related to investigation of airway involvement or to evaluate infectious and inflammatory pulmonary processes.

## INTRODUCTION

In 2020, the outbreak of coronavirus disease 2019 (COVID-19) became a worldwide emergency. Infection by the novel severe acute respiratory syndrome coronavirus 2 (SARS-CoV-2) determines a set of severe respiratory changes similar to those of severe acute respiratory syndrome.^(1,2)^

Although bronchoscopy procedures are not routinely indicated for the diagnosis of COVID-19, critically ill patients with COVID-19 admitted to intensive care unit (ICU) may require bronchoscopy for different causes during their progression.^(3,4)^

Bronchoscopy in critically ill patients with COVID-19 has been considered for evaluation and management of disease complications (removal of secretion plugs responsible for atelectasis, management of hemoptysis, evaluation of intubation airway injury, and persistent air fistulae), obtaining samples for microbiological analysis (if secondary lung infection is suspected), collecting tissue samples to aid in diagnosis of persistent inflammatory lung conditions, as well as to assist in the management of artificial airways (intubation and extubation of patients with difficult airways or to guide percutaneous tracheostomy).^(5,6)^ Bronchoalveolar lavage (BAL) collection is used to provide microbiological samples from the lower airways, and allow differential diagnosis of infectious lung disease or persistence of an inflammatory process in these cases.^(7,8)^ Transbronchial lung biopsies or biopsies of endobronchial changes may also be indicated to elucidate pulmonary or airway changes in patients on spontaneous breathing or on invasive mechanical ventilation. Mechanical ventilation is not considered a contraindication for the transbronchial biopsy collection procedure, and adds diagnostic yield in cases of negative BAL.^(9,10)^ In these situations, the presence of possible pneumothorax after the test should be monitored, with the aid of ultrasound or chest X-ray.

Many cases of air leakage in patients with COVID-19, manifested by subcutaneous emphysema, pneumomediastinum, and pneumothorax have been reported in the literature, associated with diffuse alveolar lung parenchyma damage (severe acute respiratory syndrome) or tracheal rupture.^(11)^

The causes of tracheal rupture can be divided into external post-trauma and after tracheal or spontaneous intubation.^(12)^ The pathophysiology of spontaneous tracheal rupture is not fully known and may occur after sudden increase of intratracheal pressure in patients with a local inflammatory process. Risk factors described are weakness of the tracheal wall connective tissue, such as in patients with chronic obstructive pulmonary disease or tracheomalacia, and chronic use of corticosteroids or intense coughing.^(13)^ Coronavirus disease 2019 patients present with local inflammation in the trachea and bronchi that could lead to tracheal wall weakness.^(14)^ Additionally, these are patients who may be on invasive mechanical ventilation (eventually hypoxemic), present with local mucosal ischemia (by hyperinflation of the tracheal tube cuff or inadequate local perfusion secondary to arterial hypotension), or be on corticosteroids, depending on the stage of the disease.^(1)^

Due to the scarcity of works related to bronchoscopy procedures in these patients, we described its indications and the results obtained in a reference university hospital for inpatients diagnosed with COVID-19, during the first outbreak of the disease in Brazil.

## OBJECTIVE

To describe the indications and endoscopic findings of bronchoscopies performed at a referral university hospital for inpatients diagnosed with COVID-19 during the first outbreak of the disease in Brazil.

## METHODS

A retrospective descriptive analysis was carried out with review of medical records of adult patients diagnosed with COVID-19, confirmed by reverse transcriptase polymerase chain reaction (RT-PCR) of the nasopharynx and oropharynx, associated with the clinical and radiological picture, who underwent bronchoscopy in the inpatient units of the *Instituto do Coração* (InCor) and *Hospital das Clínicas da Faculdade de* *Medicina da Universidade de São Paulo* (USP), from March 2020 to August 2020. Patients were admitted to ICU or emergency room of the hospital.

Standard flexible devices (Olympus BF-Q180 videobronchoscopes and Olympus BF-PE2 fibrobronchoscopes, Olympus Medical Systems Corporation, Tokyo, Japan) were used for the tests. Information about the indication and characteristics of the tests was obtained from the respective bronchoscopy report and the patient’s medical record. The results of the materials collected as BAL and biopsies were obtained from the electronic medical record data. All these data were stored in an Excel spreadsheet (Microsoft Corporation, Redmond, Washington, USA) for further analysis.

For patients who underwent repeated bronchoscopy tests for tracheal wall lesion follow-up, we chose to consider only the first endoscopy test in the descriptive demographic analysis. For this analysis, we used the classification in levels of involvement proposed by Cardillo et al.^(15)^ as follows: level I, with no involvement of tracheal mucosa and submucosa, with no mediastinal emphysema, and no esophageal lesion; level II, if tracheal lesion up is to the muscular layer, with subcutaneous or mediastinal emphysema, with no esophageal lesion or mediastinitis; level IIIa, if complete tracheal wall tear with mediastinal soft tissue herniation, with no esophageal injury or mediastinitis; and level IIIb, if any tracheal wall tear with esophageal injury or mediastinitis. The test results of patients with tracheal lacerations are additionally described on a separate table in the results.

The descriptive analysis consisted of calculating the simple and relative frequencies of the findings described in the bronchoscopy reports and the results of the materials collected. In the statistical analysis, comparison of means was performed for linear variables. To study categorical variables, 2x2 tables were used, and Pearson’s χ^2^ test was performed to evaluate statistical significance. Results with a probability of type I error less than or equal to 5% (p≤0.05) were considered statistically significant. The IBM SPSS, version 19.0 (IBM Corporation, Armonk, New York, USA) was used for all statistical analyses.

The study was approved by the Research Ethics Committee of the *Institution Instituto do Coração* (Incor) of the *Hospital das Clínicas* of the *Faculdade de Medicina* of the *Universidade de São Paulo*, (# 4.378.146, CAAE: 38569720.5.0000.0068).

## RESULTS

From March 2020 to August 2020, 132 bronchoscopies were performed in 103 patients diagnosed with COVID-19. The mean age was 56.1±14.5 years, with similar sex distribution (50.5% female). In 16 patients, more than one endoscopic test was performed.

The most frequent indications for bronchoscopy tests were diagnostic endoscopic evaluation and therapeutic procedures in 78.6% of cases (n=81), and collection of material for diagnosis of lung disease in 21.4% of cases (n=22), with BAL alone in 18 patients and transbronchial biopsy associated with BAL collection in four patients (Table 1).

**Table 1:** Indications for bronchoscopy in COVID-19 patients

		n (%)
Diagnostic and therapeutic bronchoscopy		81 (78.6)
	Assessment of proximal airway laceration	29 (28.2)
	Extubation assistance	16 (15.5)
	Removal of plugs and bronchial hygiene	13 (12.6)
	Tracheal cannula positioning	8 (7.8)
	Support for intubation/cannula exchange	6 (5.8)
	Hemoptysis control	3 (2.9)
	Support for percutaneous tracheostomy	3 (2.9)
	Evaluation of alveolo-pleural fistula	3 (2.9)
Collection of material		22 (21.4)
	BAL	18 (17.5)
	BAL + TBLB	4 (3.9)
Total		103 (100)

BAL: bronchoalveolar lavage; TBLB: transbronchial bronchoscopic lung biopsy.

**Figure 1:**
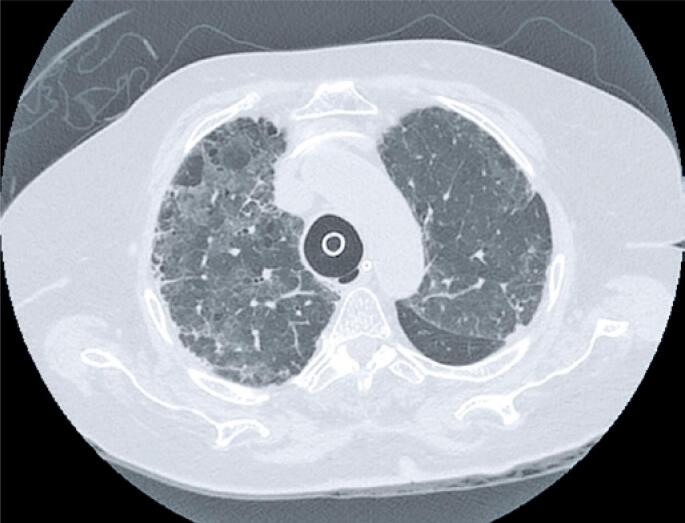
Cross-sectional computed tomography scan of COVID-19 patient showing loss of continuity of the tracheal posterior wall (tracheal tear). Note the formation of a small air sac in the lacerated region

**Figure 2:**
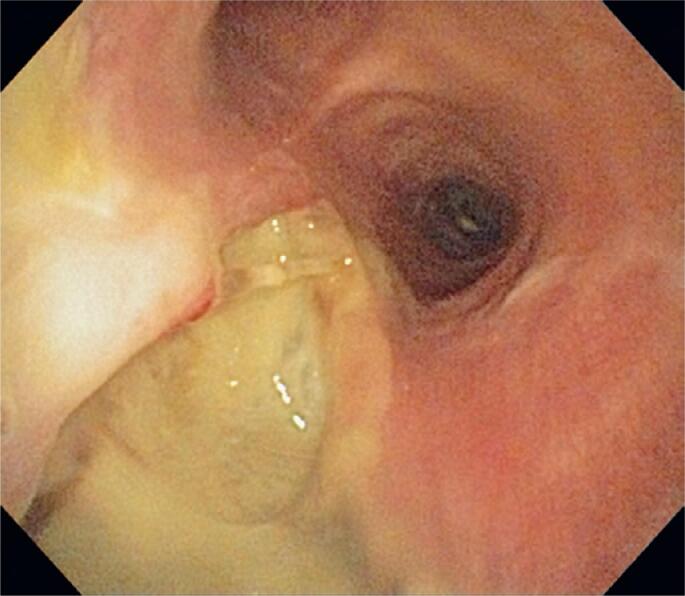
Endoscopic image showing extensive laceration of the posterior wall of the trachea with exposure of mediastinal fat and local deposition of fibrin

Regarding the diagnostic evaluation of airways, the most frequent endoscopic findings were presence of secretion or clots in 34% of cases, and acute inflammatory changes, such as diffuse laryngitis in 22.3%, and tracheal tear in 20.4%. In 27.2% of patients, no relevant bronchoscopic findings were observed. The complete description of the endoscopic findings is presented on table 2. Patients with tracheal tear (n=21) underwent more than one bronchoscopy, sequentially, for evolutive evaluation, described in table 3. Figures 1 and 2 show, respectively, examples of computed tomography scan and bronchoscopy test in patients with tracheal tear. Their mean age was similar to that of other patients (56 years). The incidence of tracheal tear was higher in female patients (71.4% in the group of patients with tear *versus* 50.5% in the general group; p=0.06), with a slightly higher mean age (57.7 *versus* 51.3 years; p=0.37). These patients had more severe alterations with greater extension and, probably for this reason, a higher mortality rate. Despite the different values, no statistically significant difference was observed in any of the parameters analyzed on table 3, due to the small number of patients considered (n=21), since the values found in each count were insufficient for a correct statistical analysis.

**Table 2:** Endoscopic findings in COVID-19 patients

Endoscopic findings		n (%)
Absence of alterations		28 (27.2)
Laryngeal changes		30 (29.1)
	Laryngitis/laryngeal edema	23 (22.3)
	Laryngeal ulcer/polyp/granuloma	6 (5.8)
	Vocal fold paralysis	1 (1.0)
Tracheal alterations		31 (30.1)
	Tracheal laceration	21 (20.4)
	Tracheitis	5 (4.9)
	Ulceration or granulation tissue (granuloma)	4 (3.9)
	Malacia	1 (1.0)
Alterations in the bronchi		39 (37.9)
	Presence of secretion or clots	35 (34)
	Mucosa laceration	3 (2.9)
	Active bleeding	1 (1.0)
Total number of alterations		100

**Table 3:** Tracheal laceration in COVID-19 patients

		Female	Male	Total n (%)
Patients		15 (71.4)	6 (28.6)	21 (100.0)
Age		57.7±12.9	51.3±12.6	55.9±12.9
Degree of involvement					
	I	-	1 (100)	1 (4.8)
	II	8 (72.7)	3 (27.3)	11 (52.4)
	IIIA	7 (77.8)	2 (22.2)	9 (42.9)
Extension (cm)		3.9±1.9	2.4±1.1	3.5±1.8
Progression					
	Death	12 (70.6)	5 (29.4)	17 (81.0)
	Discharge	3 (75.0)	1 (25.0)	4 (19.0)

Results expressed by n (%) or mean ± standard deviation.

In three patients, bronchoscopy was indicated to evaluate hemoptysis, but in only one case there was active bleeding requiring hemostasis (cold saline solution with adrenaline). In the remaining patients, only aspiration of bloody remnants from the airways was performed.

**Figure 3:**
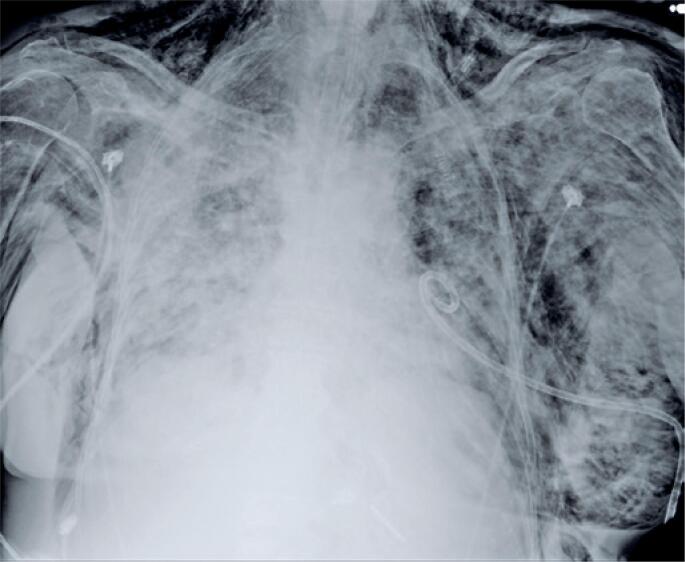
Plain chest radiograph of patient with COVID-19 and pleural fistula, with extensive thoracic and cervical subcutaneous emphysema. Presence of left pneumothorax slide and pigtail type Wayne chest drain

**Figure 4:**
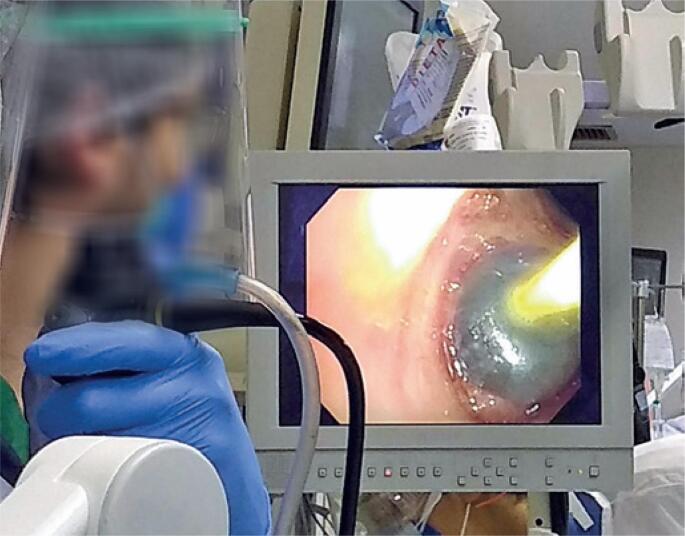
Bronchoscopy procedure showing balloon occlusion of segmental ostium during examination for pleural fistula evaluation

Patients with alveolo-pleural fistula underwent diagnostic procedures to evaluate the site of air leakage. In the same test, they were treated with bronchial occlusion by balloon catheter (Arndt-type endobronchial blocking catheter) with the aim to reduce air leakage and improve ventilatory parameters. Figures 3 and 4 show plain chest X-rays and the balloon occlusion procedure in a COVID-19 patient for identification of an alveolar-pleural fistula.

A total of 22 diagnostic procedures were performed with BAL collection. Of the 11 positive results, 40.9% were bacterial infections (seven cases with bacterial growth alone and two in association with fungi), one case was *Aspergillus sp*. infection, and one case was *Mycobacterium tuberculosis* infection. Of the bacterial infections, eight cases were by Gram-negative bacteria (six cases alone; two cases in association with fungi). The most frequently isolated Gram-negative bacteria were from the CESP group (two cases of *Enterobacter cloacae*, one case of *Serratia marcescens* and *Proteus mirabilis*), and two cases of *Stenotrophomonas maltophilia*. One patient had *Proteus mirabilis* infection and elevated galactomannan dosage, interpreted as pulmonary aspergillosis, and treated with an antifungal combination. There was only one patient with *Staphylococcus aureus*.

In this study, four patients were submitted to transbronchial lung biopsy by bronchoscopy to investigate pulmonary infiltrate with difficult resolution. These patients were on invasive mechanical ventilation and, despite positive pressure, did not have pneumothorax after the procedure. The results were consistent with organizing pneumonia. These patients had negative cultures for bacteria and fungi in the BAL samples, confirming the presence of a non-infectious inflammatory process.

No complications (hypoxemia, bleeding, and cardiac arrhythmia) were observed in this group of patients related to the endoscopic procedure. No physician who performed bronchoscopy procedures acquired the SARS-CoV-2 infection.

## DISCUSSION

Although bronchoscopy procedures were initially contraindicated for diagnosing COVID-19 due to the release of aerosols during the test, they should not be postponed in specific cases, such as in critically ill patients in emergency department or intensive care unit.^(16)^ Since it is a recently described disease, few studies have evaluated the indication of bronchoscopy in COVID-19 patients.

In this study, the main indications for bronchoscopy were related to airway procedures, ranging from evaluation of intubation trauma and aspiration of secretions to procedures with tracheal cannulas and difficult airway.

Tracheobronchial mucosal trauma following tracheal intubation is a rare but potentially fatal complication. The overall reported incidence is approximately 1:20,000 endotracheal intubations for elective procedures, although some *post-mortem* studies indicate a higher incidence in emergency cases.^(17)^ In this study, 29 patients (28.2%) had an indication to evaluate air leakage, characterized by pneumomediastinum and/or subcutaneous emphysema. In 21 of these patients (72.4%), the presence of laceration of the posterior wall of the trachea was observed. This evaluation was important in management, as, *e.g.*, to adjust the positioning of the intubation cannula to improve ventilation of the patient, or to indicate surgical repair. In general, risk factors for tracheal laceration are considered to be intubations in emergency situations with multiple attempts, lack of experience of the physician performing the intubation, advanced age, female sex (due to smaller airway caliber), and tracheal alterations, such as tracheomalacia, presence of tracheal diverticula, or tracheal bronchus.^(17)^ In this study, many cases of tracheal laceration were observed because the intubation procedure of COVID-19 patients probably involved an emergency in patients with borderline pulmonary function. As in the general literature, the prevalence was higher in female patients, with lacerations with a higher degree of involvement and more extensive, and probably because of this, there was a higher incidence of death in these patients.^(15,17)^

Torrego et al.^(7)^ evaluated 93 patients diagnosed with COVID-19 (101 bronchoscopy scans performed), with positive BAL results in 18/63 (28.6%) of cases. The prevalence of fungal infection in COVID-19 patients was variable and described as 3% to 33%.^(18)^ Fekkar et al.^(19)^ described seven cases (4.8%) in 145 patients evaluated. In this sample, of the 22 patients submitted to BAL collection for suspected pulmonary infection, two cases (9.1%) were diagnosed with pulmonary aspergillosis, showing this type of infection had a higher incidence in COVID-19 patients than in the general population.^(20)^ Unlike other studies in the literature,^(21)^ there were no cases of pulmonary pneumocystis or mucormycosis.

Although full anticoagulation is often used in patients with COVID-19, massive hemoptysis is not a frequent symptom, ranging from 0.9% to 7.3% in the literature.^(22)^ Of the patients submitted to bronchoscopy for investigation of hemoptysis, three (2.9%) had another underlying disease (diagnoses of granulomatosis with polyangiitis, cystic fibrosis, and sequelae of tuberculosis). On the other hand, bloody secretion was present in many cases, in addition to clots in the airway, possibly corresponding to a prior bleeding event.

More than 15% of patients hospitalized with COVID-19 may develop acute respiratory distress syndrome, mostly requiring mechanical ventilation and resulting in high mortality. Some studies have provided radiological evidence of developing organizing pneumonia in the late course of the disease.^(2,23)^ In the present study, four procedures with transbronchial biopsy were performed, and in all of them, the histopathological result was consistent with organizing pneumonia, with negative BAL cultures confirming an inflammatory condition and ruling out an infectious condition.

This study has some limitations. This is a retrospective study with analysis of data from medical records, which limited obtaining other data, such as mechanical ventilation parameters or clinical status at the time of bronchoscopy. The incidence of tracheobronchial alterations may be inadvertently high, because it is influenced by severity of cases, considering the hospital where the study was carried out is a unit with cases referred from other hospitals, with a large number of lacerations in the posterior wall of the trachea. On the other hand, laryngeal alterations may be underreported, since not all the patients in this study underwent laryngeal endoscopic evaluation. The analysis of BAL findings and pathogens, despite the low total number (n=22), was similar to those in the literature,^(7)^ and we should highlight the presence of fungal infection (pulmonary aspergillosis), which is a more frequent finding in COVID-19 patients.

## CONCLUSION

This study verified the application of bronchoscopy in a reference hospital for treatment of COVID-19 patients. The most frequent indications were those related to the investigation of airway involvement, or infectious and inflammatory processes of the lungs. Bronchoscopy in this group of patients provided differential diagnosis and impact on therapeutic planning. It proved to be a safe and effective method to assist in treatment of patients with COVID-19.
